# Perinatal infections – a permanent challenge

**DOI:** 10.1186/1471-2334-13-S1-O10

**Published:** 2013-12-16

**Authors:** Sorin Rugină, Irina Magdalena Dumitru, Roxana Carmen Cernat, Andra Petcu, Carmen Ilie Șerban

**Affiliations:** 1 Ovidius University, Constanța, Romania; 2 Clinical Hospital of Infectious Diseases, Constanța, Romania

## 

Perinatal infections include pregnancy associated infections, fetus infection during labor, and other types of infections, including the early postnatal and late postnatal ones that appeared in the range of one month after birth. They represent a constant threat to the health of the conceptus, fetus and newborn. Therefore their early diagnosis is fundamental in terms of establishing a fair treatment of maternal infection for prophylaxis of product conception and fetal illness and treatment of neonatal infections.

Starting from current practice realities, the authors show the need to include infectious disease specialist consultants in all periods: in antepartum, during the first trimester and occasionally in the remainder of the pregnancy and postpartum. In this way it is possible to avoid the excess diagnosis of potentially teratogenic acute infections included in the acronym TORCH during pregnancy.

